# Removal of primary nutrient degraders reduces growth of soil microbial communities with genomic redundancy

**DOI:** 10.3389/fmicb.2022.1046661

**Published:** 2023-01-24

**Authors:** Ryan McClure, Marci Garcia, Sneha Couvillion, Yuliya Farris, Kirsten S. Hofmockel

**Affiliations:** ^1^Biological Sciences Division, Pacific Northwest National Laboratory, Richland, WA, United States; ^2^Department of Agronomy, Iowa State University, Ames, IA, United States

**Keywords:** soil microbiome, primary degrader, chitin degradation, functional redundancy, synthetic community

## Abstract

**Introduction:**

Understanding how microorganisms within a soil community interact to support collective respiration and growth remains challenging. Here, we used a model substrate, chitin, and a synthetic Model Soil Consortium (MSC-2) to investigate how individual members of a microbial community contribute to decomposition and community growth. While MSC-2 can grow using chitin as the sole carbon source, we do not yet know how the growth kinetics or final biomass yields of MSC-2 vary when certain chitin degraders, or other important members, are absent.

**Methods:**

To characterize specific roles within this synthetic community, we carried out experiments leaving out members of MSC-2 and measuring biomass yields and CO_2_ production. We chose two members to iteratively leave out (referred to by genus name): *Streptomyces,* as it is predicted *via* gene expression analysis to be a major chitin degrader in the community, and *Rhodococcus* as it is predicted *via* species co-abundance analysis to interact with several other members.

**Results:**

Our results showed that when MSC-2 lacked Streptomyces, growth and respiration of the community was severely reduced. Removal of either *Streptomyces* or Rhodococcus led to major changes in abundance for several other species, pointing to a comprehensive shifting of the microbial community when important members are removed, as well as alterations in the metabolic profile, especially when Streptomyces was lacking. These results show that when keystone, chitin degrading members are removed, other members, even those with the potential to degrade chitin, do not fill the same metabolic niche to promote community growth. In addition, highly connected members may be removed with similar or even increased levels of growth and respiration.

**Discussion:**

Our findings are critical to a better understanding of soil microbiology, specifically in how communities maintain activity when biotic or abiotic factors lead to changes in biodiversity in soil systems.

## Introduction

Microbial metabolism in soil is critical to a number of functions related to promotion of plant growth in the face of environmental or pathogen stress (Hannula et al., 2020; Tian et al., 2020) and to the cycling of carbon and other nutrients (Gougoulias et al., 2014; Scarlett et al., 2021). As carbon (C) enters the soil (either through plant exudates, plant litter or other means) there are multiple microbial metabolic pathways that support anabolic (cell growth and replication, increases in biomass) or catabolic (CO_2_ respiration) activity. How microbial species or communities partition incoming carbon into either biomass or respiration pathways defines their carbon use efficiency (CUE; Li et al., 2014; Saifuddin et al., 2019). CUE can be examined in several ways, including at the ecological and population scale. When examining CUE with respect to communities it is defined as the gross biomass production per unit substrate taken up over short time scales. This definition only considers the uptake, not the subsequent loss of C as a function of microbial necromass and exudates (Geyer et al., 2016) but does factor in C lost through respiration as CO_2_.

When quantifying the functions carried out by the soil microbiome, or when analyzing values such as CUE, viewing communities as the sum of individual members can lead to unidentified sources of variation. This is due to the fact that it is interactions between microbial species that define their metabolism and CUE values (Pan et al., 2021) not merely the sum of the individual community members in isolation (Saleem et al., 2019). The interaction network that emerges as these species exchange nutrients, compete for resources, or shift their phenotypes leads to certain species emerging as keystone members of the community. Microbial keystone species are defined as taxa with a large number of interactions with other members of the community and/or those that exert a large effect on the community structure or functioning (Banerjee et al., 2018). Keystone taxa may be those that carry out critical ecological functions in the community and, as a result, when they are lost, the community may show much less stability and activity (Xun et al., 2021).

Keystone species may be especially likely to emerge in small communities, with less opportunity for redundancy, when they are provided with complex carbon or nitrogen sources that not all community members can metabolize equally (Liu et al., 2021). The reduced size of these communities, both in terms of number of species and number of available metabolic processes, means that only a few members can play a role as keystone members or primary degraders in the community, and if these members are lost the community suffers. Keystone members are also defined by the environment. Under conditions with varied nutrient sources that many members can metabolize keystone members may be few. However, under conditions with a small number of nutrient sources, especially those that may be energetically expensive to metabolize, keystone members may be more important (Duan et al., 2022). Chitin is one such nutrient source that often promotes interspecies interactions when degraded. Previous work has shown that in a monoculture of a chitin degrading species only a small subset of cells actually produce chitinase and chitin breakdown products (chiefly N-acetyl-glucosamine, NAG) with the remaining cells subsisting off these breakdown products (Baty III et al., 2000a,b; Zhou et al., 2020). In addition, chitinase enzymes are often not cell associated (Itoh et al., 2013; Lobo et al., 2013; Johnson-Rollings et al., 2014) meaning the breakdown products can be more easily shared. In accordance with this, species have been found that contain NAG importers with no corresponding chitinase gene, suggesting they obtain NAG through the breakdown of chitin carried out by other species (Keyhani and Roseman, 1997). As chitin is an abundant C and nitrogen source in soil, keystone species in these communities may center around chitin breakdown with the primary chitin degraders in a community occupying the keystone positions. Even in communities that may have functional redundancy at the genomic level, the possibility for keystone species still exists. This is because genomic potential is not a direct predictor of phenotypic expression (Li et al., 2019). Environmental factors, including interspecies interactions, can alter the expression of a gene or enzyme, especially if this enzyme may be energetically unfavorable to synthesize (McClure et al., 2022).

Primary degraders and keystone species play important roles in soil microbiomes but the inherent complexity of this site, with thousands of species and metabolites and potentially millions of interactions, means that evaluating keystone species and how they may drive CUE can be difficult in the native soil. Recently, we sought to reduce this complexity through the generation of a model soil consortium (MSC). Previous work has developed MSC-1, a naturally evolved community of ~25 species (McClure et al., 2020) and MSC-2, a synthetic community of eight species isolated from MSC-1 (McClure et al., 2022). Within MSC-2 we identified two potential keystone species: a *Rhodococcus* species was found to be highly connected to other species in a co-abundance network of MSC-1 (McClure et al., 2020) while a *Streptomyces* species was found to be the primary, and perhaps only, chitin degrading species within the community (McClure et al., 2022). Here, we cultured MSC-2 lacking each of these species in turn and compared it to the complete MSC-2 community with reference to biomass accumulation, respiration, and ultimately CUE. We also collected and analyzed data showing the resulting community make up (amplicon analysis) and metabolite environment. Our results show how synthetic, representative microbial communities respond to the absence of different varieties of potential keystone species. These conclusions in turn can reveal how natural soil communities, and their C and nutrient cycling properties, may respond when primary degraders increase or decrease their abundance in response to changing environments.

## Materials and methods

### Growth of MSC-2 and MSC-2 subsets with chitin

A detailed description of MSC-2 is in our previous publication (McClure et al., 2022) but briefly this community is comprised of eight strains of common soil bacteria: a *Streptomyces* sp., *Neorhizobium tomejilense*, *Dyadobacter fermentans*, *Sphingopyxis fribergensis*, *Ensifer adhaerens*, *Variovorax beijingensis*, *Sinorhizobium meliloti* and a *Rhodococcus* sp. For simplicity each member is referred to by its genus name. To set up cultures of the complete MSC-2 community or of subcommunities lacking certain members, each MSC-2 strain was plated on an R2A agar plate and incubated at 20°C for 3 days. Following incubation, growth was collected from each plate and used to set up a 5 mL liquid culture, one for each of the eight members of MSC-2. These cultures were shaken at 150 rpm at 20°C overnight in 50 mL breathable falcon tubes containing R2A medium with 100 ppm chitin. Cells were then spun down for 5 minutes at 5,000 g, washed in R2A and spun down again before being resuspended in 5 mL of fresh R2A. Resuspended cultures were added to 95 mL of R2A with 100 ppm chitin in a 500 mL flask and shaken at 150 rpm at 20°C overnight. After overnight growth, cultures were spun down as above, washed with M9, spun down again, and resuspended in 10 mL of M9. M9 consisted of 12.8 g/L Na_2_HPO_4_-7H_2_O, 3 g/L KH_2_PO_4,_ 0.5 g/L NaCl, 1 g/L NH_4_Cl, 4 mM MgSO_4_, 100 uM CaCl_2_, 1 mg/L biotin, 1 mg/L thymine, 1 mL/L of 1,000x wolfs trace minerals (comprised of the following per 1 l: HCl 1 mL, Na_4_EDTA (tetrasodium) 0.5 g, FeCl_3_ 2 g, H_3_BO_3_ 0.05 g, ZnCl_2_ 0.05 g, CuCl_2_ 0.03 g, MnCl_2_ 0.05 g (NH_4_)_2_MoO_4_ 0.05 g, AlK (SO_4_) 0.05 g, CoCl_2_ 0.05 g, NiCl_2_ 0.05 g) and 5 mL/L of vitamin solution (comprised of the following per 1 l: 10 mg each of Niacin, Pantothenate, Lipoic Acid, *p*-Aminobenzoic Acid, Thiamine B_1_, Riboflavin B_2_, Pyridoxine B_6_ and Cobalamin B_12_ and 4 mg each of Biotin and Folic Acid). Additional biotin and thymine were added as that was found to lead to better growth of species.

Specific volumes of each strain were then collected and combined in a 50 mL falcon tube aiming for a final volume of 10 mL. Volumes were chosen so that cell numbers would be equal between all eight members using previously collected O.D. and colony forming unit (C.F.U.) data. Three different communities were made through combinations of MSC-2 members. One community consisted of all eight members of MSC-2 (MSC-2 Complete), one community consisted of all members except *Rhodococcus* (MSC-2-R), and one community consisted of all members except *Streptomyces* (MSC-2-S). The O.D._600_ of each of the three communities was then adjusted by dilution to between 0.08–0.12 and chitin was added to a final concentration of 500 ppm. From these communities 9 mL was pulled and used to set up replicate tubes of each community (5 replicates of MSC-2 Complete, 5 replicates of MSC-2-R and 5 replicates of MSC-2-S, each containing 9 mL). Cultures were grown in 40 mL amber borosilicate tubes with snap top septa cap with shaking (150 rpm) at 20°C. Cell counts were collected every 24 h using an 11th edition Agilent Novocyte flow cytometer. For all samples chitin was allowed to settle for 5 min before cells were counted. CO_2_ measurements were taken every day starting after the first 24 h using an EGM-4 environmental gas monitor. After 7 days of growth cell pellets were collected for 16S amplicon analysis and supernatants were collected for metabolomics analysis.

### 16S amplicon analysis

DNA was extracted from bacterial cell pellets using the ZymoBIOMICS DNA Miniprep Kit. 16S sequencing was carried out by GENEWIZ (South Plainfield, NJ). The proprietary workflow at GENEWIZ effectively amplifies the three variable regions of the16S rRNA (V3, V4, and V5) using paired end read Illumina technology. Following sequencing, the two sequences of each read pair were merged according to overlapping sequences. The read merge is deemed to be successful only if the overlapping sequence is least 20 bp long. After merging, undetermined bases (N) were removed from the resulting sequence and primer and adapter sequences were removed. The 5′ and 3′ bases with Q score lower than 20 were also removed. The sequences obtained were then aligned to UCHIME ‘Gold’ database to identify and remove chimera sequence. Sequences passing this filtering step are deemed as clean data ready for analysis. Clustering of OTUs and taxonomic identification was carried out *via* QIIME2 (Bolyen et al., 2019).

### Metabolomics analysis

Metabolomic analysis of MSC-2 and MSC-2 subsets was carried out by gas chromatography mass spectrometry (GC–MS) as previously described (Cao et al., 2021). Samples were grouped into blocks and randomized for each experiment. GC–MS raw data processing was done with the Metabolite Detector software and metabolites were identified by matching experimental spectra and retention indices to an augmented version of FiehnLib (Kind et al., 2009). In addition, the NIST 14 GC–MS library was used to cross-validate spectral matching scores obtained by using the Agilent library and to provide identifications of unmatched metabolites. After removing outlier samples, four biological replicates were analyzed for MSC-2 and MSC-2-R and five biological replicates for MSC-2-S. Metabolomics data was processed using MetaboAnalyst 5.0 (Pang et al., 2022). The data was normalized by sample median and log transformed. Significant features were identified by a one-way ANOVA (value of p threshold >0.05) followed by the Tukey’s HSD post-hoc analyses. The autoscaled metabolite abundances for significant and annotated metabolites was visualized as a heatmap and hierarchical clustering of the metabolites was done using Euclidean distance measure and Ward clustering algorithm. Metabolites in the MSC-2-S or MSC-2-R communities were then compared to the complete MSC-2 and metabolites that shifted their abundance between communities with an adjusted *p*-value (by Benjamini-Hochberg) of less than 0.05 were the focus of our analysis.

## Results

### Respiration of MSC-2 and MSC-2 subsets

The complete MSC-2 community consists of eight members (referred to by their genus name for simplicity): *Streptomyces*, *Neorhizobium*, *Dyadobacter*, *Sphingopyxis*, *Ensifer*, *Variovorax*, *Sinorhizobium* and *Rhodococcus*. Previous work by our group has found that *Rhodococcus* occupies a central and important position in this community (McClure et al., 2020). This suggests that *Rhodococcus* may interact with many other species and its removal may disrupt key interaction networks. Additional work by our group has also found that *Streptomyces* is the primary degrader of chitin in this community (McClure et al., 2022). However, other species can grow using chitin as the sole C source and thus may act as chitin degraders when the primary degrader is removed. To explore the role of each different type of potential keystone species in the context of community growth and CUE (biomass accumulation vs. CO_2_ production) we examined respiration rates and cell counts of three different communities: the complete 8-member MSC-2 community, MSC-2 lacking *Rhodococcus* (MSC-2-R) and MSC-2 lacking *Streptomyces* (MSC-2-S) under conditions where each of these communities was grown using chitin as the sole C source.

**Figure 1:**
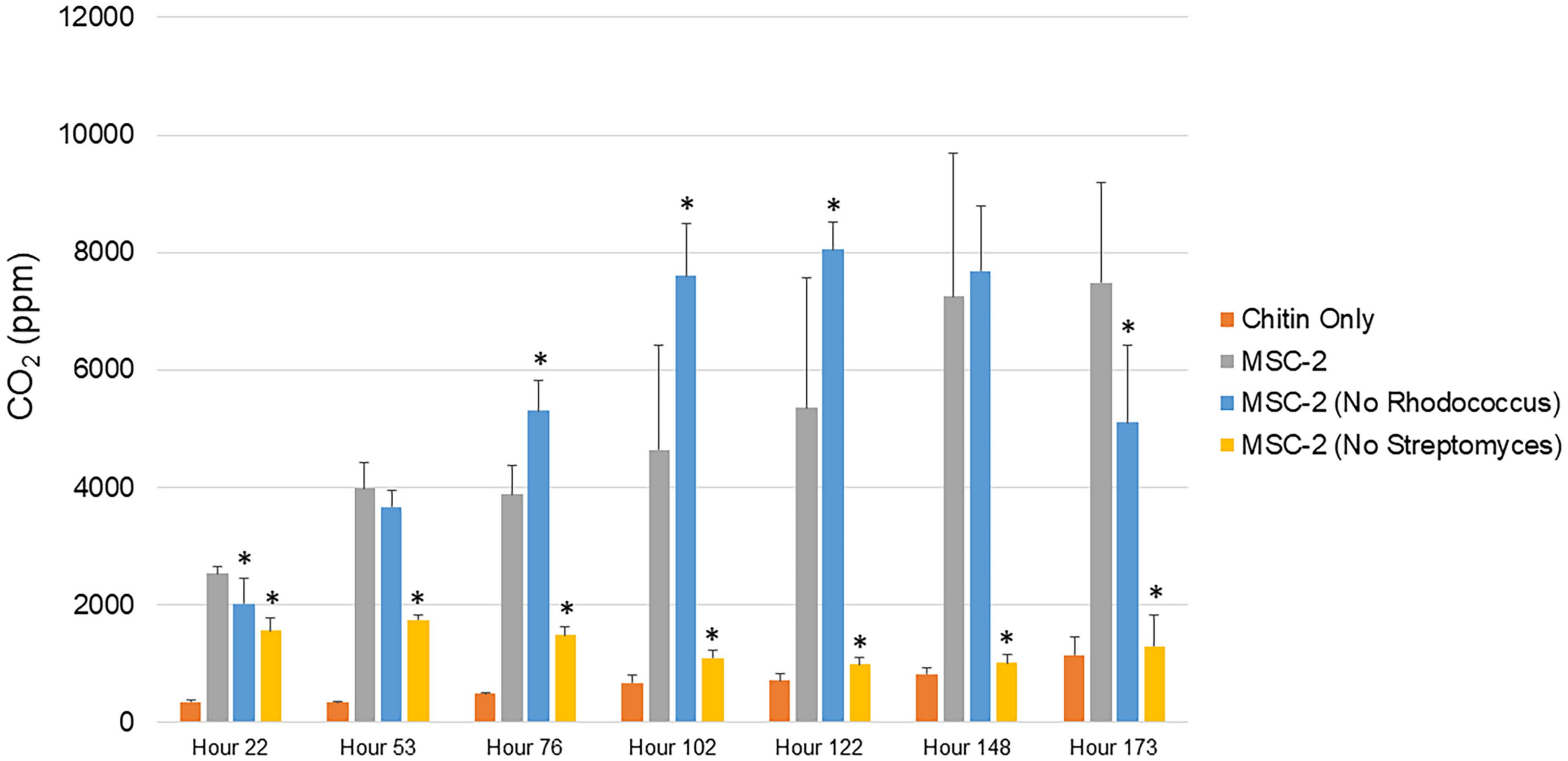
Respiration of MSC-2 and MSC-2 subset communities during chitin growth. CO_2_ levels in parts per million are shown on the y-axis and timepoints ranging from 22 to 173 h are shown on the x-axis. Orange bars are uninoculated samples containing only chitin, grey bars are complete MSC-2 communities, blue bars are MSC-2-R communities (lacking *Rhodococcus*), and yellow bars are MSC-2-S communities (lacing *Streptomyces*). Error bars indicate standard deviation and asterisks indicate that the community is statistically different from the MSC-2 community at that timepoint with a *p*-value of less than 0.05 using a Students *t*-test.

Approximately 24 h after the start of growth MSC-2-S and MSC-2-R have significantly lower CO_2_ production compared to MSC-2 (Figure 1). By 53 h after the start of growth, the difference in CO_2_ production between MSC-2-S and MSC-2 becomes more pronounced and the difference between MSC-2 and MSC-2-R becomes less as the MSC-2-R community begins to increase respiration. This trend continues to a timepoint collected 76 h after the start of growth; by this point MSC-2-R is producing more CO_2_ compared to MSC-2 and this difference is statistically significant. Respiration levels of the three communities continue in this way until 173 h after the start of growth when CO_2_ production by MSC-2-R is beginning to drop (continuing a decline started at 122 h) while the respiration rates of the complete MSC-2 community have continued to rise and are just beginning to peak. At the final timepoint of 173 h MSC-2-R has again fallen below MSC-2 in CO_2_ production with this difference reaching statistical significance. Through this entire respiration assay the MSC-2-S communities show a very slight early increase in CO_2_ production, but consistently remain far below both MSC-2 and MSC-2-R communities, in some cases showing CO_2_ levels that are barely above background (collected from non-inoculated control tubes containing chitin only). Compared to MSC-2 communities, MSC-2-R communities show a faster rise in CO_2_ production followed by a similarly early fall while MSC-2-S communities show consistently far less respiration.

### Biomass of MSC-2 and MSC-2 subsets

**Figure 2:**
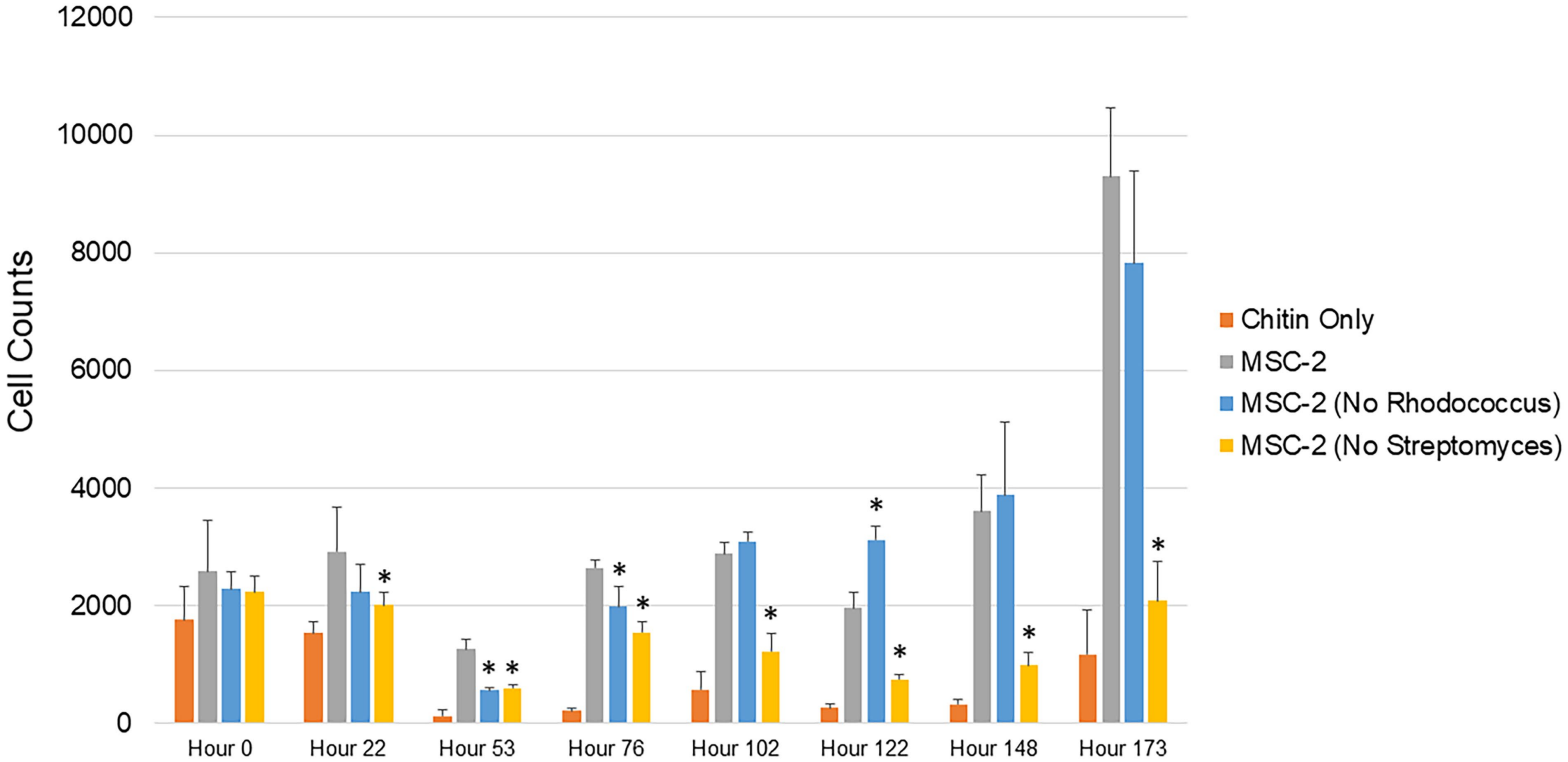
Cell counts of MSC-2 and MSC-2 subset communities during chitin growth. Cell counts are shown on the y-axis and timepoints ranging from 0 to 173 h are shown on the x-axis. Orange bars are uninoculated samples containing only chitin, grey bars are complete MSC-2 communities, blue bars are MSC-2-R communities (lacking *Rhodococcus*), and yellow bars are MSC-2-S communities (lacing *Streptomyces*). Error bars indicate standard deviation and asterisks indicate that the community is statistically different from the MSC-2 community at that timepoint with a *p*-value of less than 0.05 using a Student’s *t*-test.

Production of CO_2_ represents one possible fate for C obtained through degradation of chitin. An alternative path for C is to be incorporated as biomass of microbial cells. To explore this, we also used cell counting to determine the number of bacterial cells at timepoints similar to the CO_2_ assays shown above (Figure 2). The overall trend of these biomass assays mirrored the CO_2_ assays. Cell counts in MSC-2-S communities were consistently lower than MSC-2, in most cases significantly so, this is despite cell counts at the start of the experiment showing the same amount for each community, indicating that they all begin at the same “starting line.” Cell counts for all communities showed a drop at 53 h but began to generally show increases after that. By 102 h after the start of the growth assay the MSC-2-R community showed higher cell numbers compared to MSC-2. However, by the end of the assay, similar to the respiration levels, the cell counts in the MSC-2-R community had dropped below those of MSC-2 (though not significantly so). One major difference between the cell count assay and the CO_2_ assay was at the end point of the experiment. Cell counts continued to increase for both MSC-2 and MSC-2-R even at 173 h. For respiration, in both communities, CO_2_ production peaked at 148–173 h for MSC-2 and for MSC-2-R actually began to fall at 173 h (Figure 1). The collection of both respiration and biomass assays allow us to characterize CUE patterns for the communities across time. Early in the growth experiment CUE rates are low, respiration is dominant, and cell counts levels drop slightly. By 102 h after the start CUE rates begin to rise as biomass levels increase. By the end of the experiment at 173 h, CUE levels have maximized with biomass very high and respiration beginning to fall (MSC-2-R) or stabilizing (MSC-2). However, when viewing both respiration or biomass accumulation communities lacking *Streptomyces* (MSC-2-S) show far lower values across almost all timepoints when compared to either MSC-2 or MSC-2-R communities.

### Amplicon analysis of MSC-2 and MSC-2 subsets

**Figure 3:**
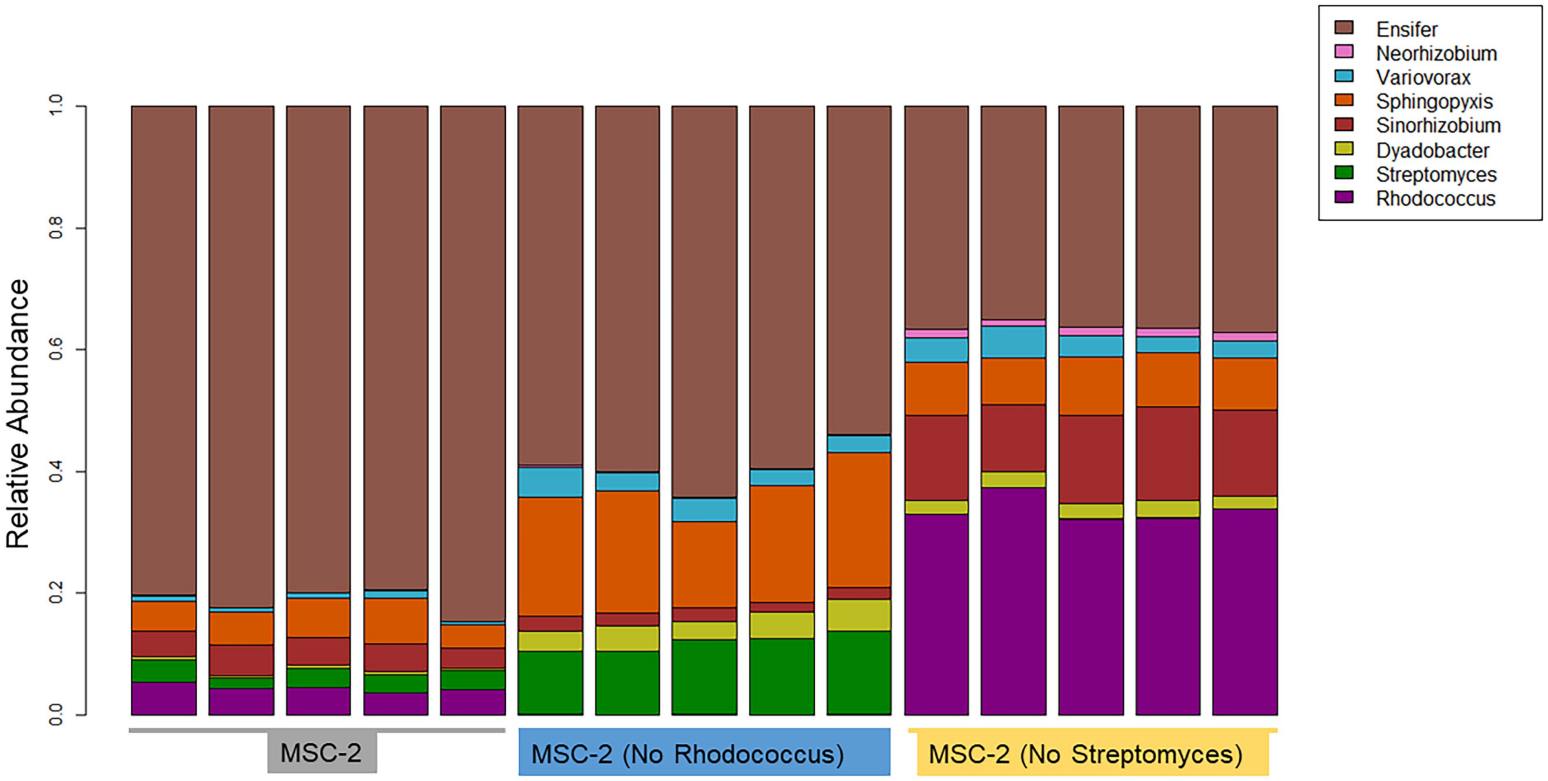
Taxonomic bar charts of MSC-2 members after chitin growth in MSC-2, MSC-2-R and MSC-2-S. Relative abundance is shown on the y-axis. MSC-2, MSC-2-R and MSC-2-S communities are grouped together on the *x*-axis. Color scheme assigned to each species is shown on the upper right.

16S amplicon analysis was used to query the relative abundance of each species at the conclusion of the 7-day growth assay (Figure 3). In the MSC-2 community *Ensifer* was the dominant community member, comprising approximately 80% of the community with *Rhodococcus*, *Sphingopyxis*, *Sinorhizobium* and *Streptomyces* comprising most of the remaining 20%. When *Rhodococcus* was removed from the community, in MSC-2-R samples, *Ensifer* dropped in abundance from ~80% to ~60%. Table 1 shows the fold changes of other members of MSC-2 and how their abundances shift as a function of loss of *Rhodococcus*. Aside from *Ensifer, Sinorhizobium* also showed a drop in abundance of nearly 2-fold. This drop in abundance for *Ensifer* and *Sinorhizobium* was concurrent with a relative increase in abundance for the other five members of this community. Of this *Dyadobacter* showed the greatest improvement with an approximately 10-fold increase in relative abundance. However, even with this increase *Dyadobacter* remained a low abundant member similar to what we have seen before (McClure et al., 2022) *Streptomyces* was more abundant and showed an increase of approximately 4-fold increase, going from ~3% to 10% in the absence of *Rhodococcus*. *Variovorax* also showed an increase in abundance as well.

**Table 1:** Fold changes and *p*-values of member relative abundances in MSC-2-R and MSC-2-S communities.

MSC-2 member	Fold change		*p*-value	
	MSC-2-R/MSC-2	MSC-2-S/MSC-2	MSC-2-R vs. MSC-2	MSC-2-S vs. MSC-2
*Rhodococcus*	NA	9.07	NA	3.01E-09
*Streptomyces*	4.56	NA	8.91E-06	NA
*Dyadobacter*	10.71	6.81	2.04E-04	5.07E-07
*Sinorhizobium*	0.52	3.78	5.58E-04	9.86E-07
*Sphingopyxis*	3.84	1.84	1.10E-04	3.52E-04
*Variovorax*	4.49	5.18	6.80E-05	6.77E-04
*Neorhizobium*	2.18	15.84	5.22E-04	1.50E-07
*Ensifer*	0.82	0.53	4.70E-04	2.82E-08

We next compared the complete MSC-2 community to the community lacking *Streptomyces* (MSC-2-S). Without *Streptomyces* the community was much more even compared to the complete MSC-2 community and there was an even greater drop in *Ensifer*, the highly abundant member of MSC-2 (when compared to MSC-2-R). In MSC-2-S communities *Ensifer* dropped to ~36% with all other species increasing their relative abundance. Of these *Neorhizobium* had the biggest gain, going from 0.9% to more than 4%, a 15-fold increase. Interestingly, *Neorhizobium* did not show this magnitude increase in MSC-2-R samples even though *Ensifer* dropped there as well suggesting that the increase in *Neorhizobium* in MSC-2-S samples is due to lack of *Streptomyces*, not loss of *Ensifer*. *Rhodococcus* was the species that showed the next largest increase compared to MSC-2. *Rhodococcus* increased by more than 9-fold, going from ~4% to ~33% of the total community. *Sinorhizobium* also showed a ~ 3.7-fold increase in abundance. Like *Neorhizobium* this increase for *Sinorhizobium* was a very different response compared to that seen in the MSC-2-R community suggesting that it was lack of *Streptomyces*, not just a drop in *Ensifer* that led to this increase for *Sinorhizobium*. Other species also showed increases in abundance but their response in MSC-2-R and this community, MSC-2-S, were similar. All changes in abundance described in this section are statistically significant with a *p*-value of less than 0.05. It should be noted here that due to the nature of 16S sequencing the drop in *Ensifer* may open up more room on the sequencing flow cell allowing other species to show relative increases even if they are the same abundance. Species that show different responses in MSC-2-R and MSC-2-S communities, both of which lack *Ensifer*, are of interest as their response is likely not due to *Ensifer* abundance shifts but to true responses to the changing community. A PCA plot of all samples also showed clear delineation of the communities and that the variation between replicates was much smaller in the MSC-2-S community compared to the MSC-2 or MSC-2-R community (Supplementary Figure S1).

### Metabolomic analysis of MSC-2 and MSC-2 subsets

**Figure 4:**
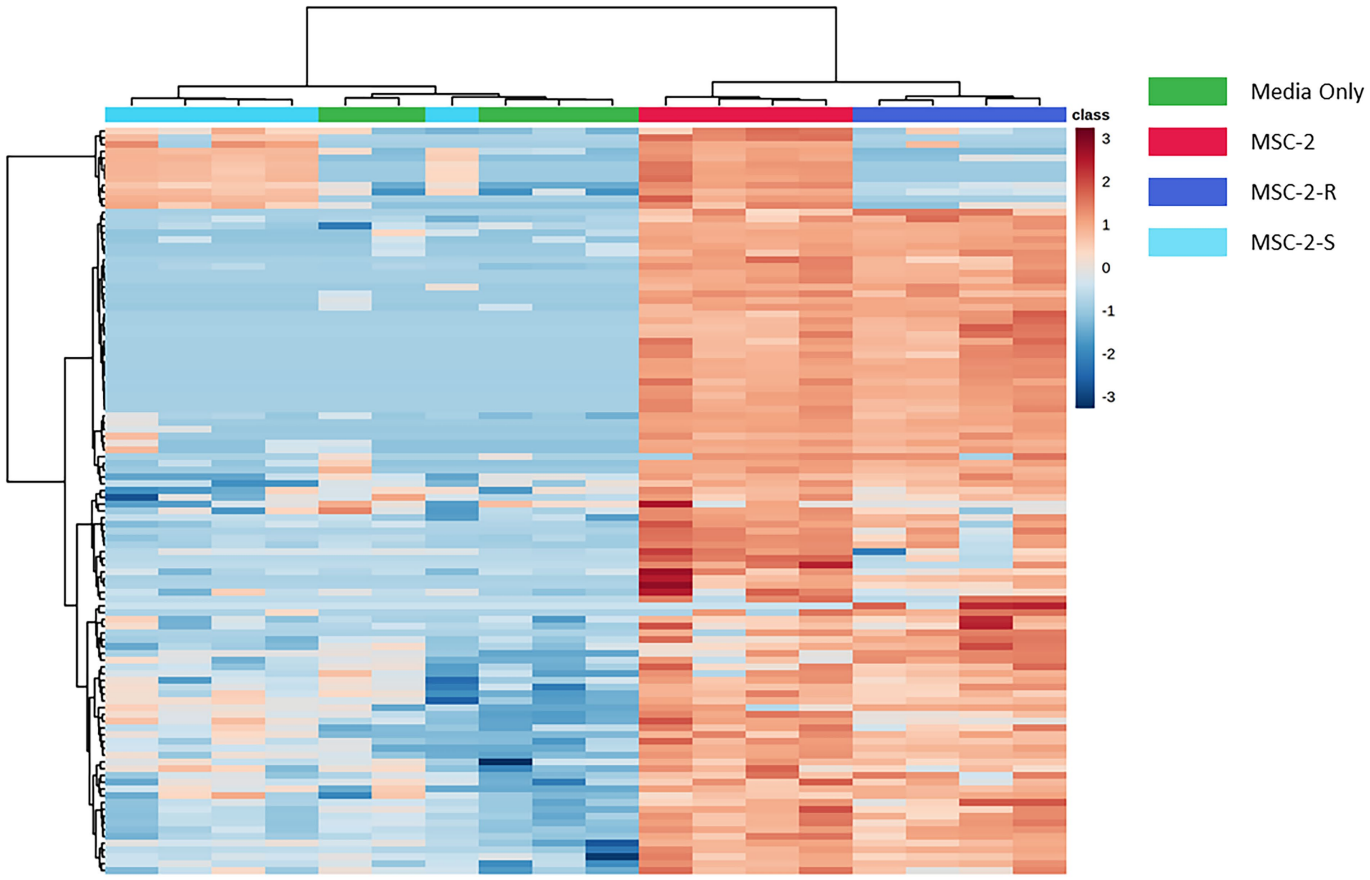
Heatmap of differentially abundant metabolites in MSC-2 communities, subsets and media controls. Both rows and columns are hierarchically clustered based on similarities in samples or metabolites, respectively, and color schemes indicate the sample type. Blue indicates that the metabolite is less abundant compared to all samples and red indicates that it is more abundant compared to all other samples. All metabolites included in the heatmap are statistically significant when comparing MSC-2-R to MSC-2 or when comparing MSC-2-S to MSC-2.

We next examined the metabolic profile of MSC-2 and each of the subsets. Here, results were somewhat different from amplicon analysis. While MSC-2-R and MSC-2-S were both distinct from MSC-2 (Supplementary Figure S2) a closer look at how metabolites differed shows that MSC-2-R was much more similar to MSC-2 compared to MSC-2-S. In fact, the MSC-2-S community was more similar to media blanks than either MSC-2 or MSC-2-R suggesting that without *Streptomyces* the metabolite profile of the environment shows the least change from starting conditions. Changes that did occur in the MSC-2-S community were, in many cases, the exact opposite for statistically significant metabolites compared to either MSC-2-R or the complete MSC-2 (Figure 4). We also looked in more detail at which specific metabolites were of higher or lower abundance in the MSC-2-R and MSC-2-S communities compared to the complete MSC-2. When comparing MSC-2-R to the complete MSC-2, 42 metabolites were differentially abundant. When comparing MSC-2-S to the complete MCS-2, 107 metabolites were differentially abundant. Table 2 shows some of the identified metabolites that were differentially abundant between the complete MSC-2 and the subcommunities. Focusing on metabolites that showed different fold changes in the MSC-2-R and MSC-2-S communities identified trehalose, adenine, succinic acid, N-carbamyl-L-glutamic acid, caffeic acid and mannobiose. Trehalose and succinic acid showed a decrease in communities lacking *Rhodococcus* and an increase in communities lacking *Streptomyces*. Adenine, N-carbamyl-L-glutamic acid, caffeic acid and mannobiose showed an increase in communities lacking *Rhodococcus* and a decrease in communities lacking *Streptomyces.*

**Table 2:** Fold changes of metabolites in MSC-2-R and MSC-2-S communities.

Metabolite	Fold change MSC-2-R/MSC-2	Fold ChangeMSC-2-S/MSC-2
Citramalic acid	0.491	0.035
Palmitic acid	1.254	2.155
Trehalose	0.922	3.176
Stearic acid	1.350	2.027
4-methyl-5-thiazoleethanol	1.607	2.902
3,4-dihydroxybenzoic acid (protocatechuic acid)	1.538	2.827
Adenine	1.527	0.024
Lactic acid	1.235	2.401
L-pyroglutamic acid	0.588	0.871
Succinic acid	0.920	1.491
2-isopropylmalic acid	0.073	0.002
N-carbamyl-L-glutamic acid	2.199	0.513
Caffeic acid	1.539	0.038
Porphine	1.224	2.512
5-hydroxymethyl-2-furoic acid	1.284	2.192
Mannobiose	2.454	0.455

## Discussion

Within microbial communities, certain species often occupy important positions as primary degraders or keystone members. Here we explored how a defined community may respond when different kinds of keystone members (those highly connected to other members or those acting as primary degraders of nutrients) are removed. We found that when a keystone member defined by connectedness (*Rhodococcus*) was removed there was no negative effect on community growth or respiration, in fact the community did somewhat better. In contrast, when a keystone member defined by a chitin degradation phenotype (*Streptomyces*) was removed there was a severe loss of growth and respiration in the resulting community. Previous work (McClure et al., 2022) has strongly suggested that *Streptomyces* is the primary chitin degrader in the MSC-2 community. However, this same earlier study also showed that additional species are able to grow on chitin (as measured by increases in O.D.) even in monoculture. Despite redundancy in genomic potential and functional assays carried out in monoculture, functional redundancy (as measured by growth and respiration) was not expressed in the community context when the primary chitin degrader was removed. Why do these other species not emerge as alternative primary degraders and keystone members in the absence of *Streptomyces* to drive community growth to levels seen in the complete MSC-2 community? It may be that a without *Streptomyces* no species is able to act as a chitin degrader and that the minimal growth of the MSC-2-S community reflects necromass metabolism. While we do see that many of the MSC-2 species are able to grow on chitin in monoculture, phenotypes expressed in a community often do match those seen in monoculture (Heyse et al., 2019; Zuñiga et al., 2019), something we have also seen in our previous publications examining MSC-2 (McClure et al., 2022). Inhibitions between species may prevent one of the remaining chitin degrading members from expressing this phenotype and the community as a whole is stuck in its initial state (Geesink et al., 2018). This possibility is supported by the fact that the MSC-2-S community is still relatively even after 8 days, suggesting minimal growth and cell turnover and the fact that the metabolic profile is very similar to the initial growth media. Alternatively, some species may act as chitin degraders but they are simply not as efficient as *Streptomyces* so the community takes longer to reach high biomass levels. This is supported by our cell count measurements (Figure 2) showing that between hours 122 and 173 the CUE of the MSC-2-S community is high, biomass levels are increasing, and respiration is low, and that if the experiment had continued the MSC-2-S community may reach levels seen with the complete MSC-2. Our previous work (McClure et al., 2022) and others have shown variation among bacteria that can degrade chitin with regard to the rate that this takes place and how this drives subsequent growth (Brankatschk et al., 2011; Köllner et al., 2012).

However, we propose here that the lack of growth we see in the MSC-2-S community is related to the identity of the emerging chitin degrader in a community without *Streptomyces*. Our hypothesis is that without *Streptomyces*, *Rhodococcus* acts as the primary chitin degrader. However, the interactions that *Rhodococcus* has with other MSC-2 species means that chitin breakdown products generated by *Rhodococcus* are not shared efficiently with other MSC-2 members, or at least not as efficiently as when *Streptomyces* is the primary chitin degrader. This leads to slower overall growth of the MSC-2-S community. Multiple lines of evidence support this conclusion. First, in a version of MSC-2 lacking *Rhodococcus* the community actually grows better, as measured by biomass accumulation and respiration, compared to the complete MSC-2 community that contains *Rhodococcus*. This suggests possible antagonistic interactions that *Rhodococcus* may have with other species, possibly related to sharing of chitin breakdown products. The idea that *Rhodococcus* may be in competitive rather that cooperative relationships is also supported by our previous network analysis showing negative co-abundances between *Rhodococcus* and other MSC-2 members including *Streptomyces* and *Variovorax* (McClure et al., 2020). Our observation here that *Streptomyces* and *Variovorax* actually increase their abundance when *Rhodococcus* is removed (Figure 3) supports these previous observations and our hypothesis here. A second line of evidence is related to the fact that *Rhodococcus* grows extremely well on chitin in monoculture (McClure et al., 2022), better than any other member of MSC-2, showing that it is able to make the most efficient use of chitin as a C source. Third, *Rhodococcus’* relative abundance is much higher in a community lacking *Streptomyces* (MSC-2-S) than in the complete MSC-2 community. In the complete MSC-2 community *Ensifer* is the dominant member, likely due to its large fundamental niche as we describe in a previous publication (McClure et al., 2022), but in the MSC-2-S community *Ensifer* is still abundant (~36% of the community), but it is virtually tied in abundance with *Rhodococcus* (~33% of the community). Without *Streptomyces* in the community to degrade chitin, *Rhodococcus* steps in as a chitin degrader. It’s efficient use of chitin as a C source (seen in our previous publication (McClure et al., 2022)) means that the abundance of *Rhodococcus* increases. However, competitive interactions with other species (seen in our previous species co-abundance networks (McClure et al., 2020)) means that *Rhodococcus*-derived chitin breakdown products are not shared as efficiently and the community as a whole exhibits slow growth. This may take place even though chitin breakdown happens extracellularly, and breakdown products are at least in theory available to the whole community. *Rhodococcus* may possess cell associated chitinase enzymes that are not free floating, as has been seen with bacteria (Itoh et al., 2013). If this is the case, then NAG and other chitin breakdown products would be more available to *Rhodococcus* cells (since breakdown happens just adjacent to the cell) compared to other MSC-2 members. All of this means that even though *Rhodococcus* does well, its success does not translate to high biomass accumulation or respiration of the whole community.

The identity of some bacterial species as ‘selfish’, meaning they do not share breakdown products with other members, has been found in other systems (Reintjes et al., 2019, 2020; Manna et al., 2022). Many of these previous studies have found that a “selfish” phenotype is related to the expression of excreted extracellular enzymes that process C sources. Chitinase is known to be an extracellular enzyme; but while its excretion has been demonstrated in multiple studies for *Streptomyces* (which likely shares chitin breakdown products in MSC-2) (Joo, 2005; Narayana and Vijayalakshmi, 2009; Ray et al., 2019) it is less well known if this is the case for *Rhodococcus*. If *Rhodococcus* chitinase enzymes are extracellular but remain cell associated (and are not fully excreted) this may mean that *Rhodococcus* would not share chitin breakdown products as efficiently as *Streptomyces*. Supporting *Streptomyces* as a sharing species our analysis identified mannobiose as a metabolite that was much more abundant when *Streptomyces* was present vs. in a community lacking *Streptomyces*. This C source has been shown to be produced by *Streptomyces* and consumed by *Rhodococcus* (Ohashi et al., 2021).

Our identification here of a potential keystone species whose removal leads to better community growth and may be acting as a selfish member emphasizes that highly connected members are not necessarily those that promote community growth but only those that have an effect on community structure and functioning. In some cases, removing a highly connected member may be good for a community if that member has negative interactions with other community members, especially other keystone members. These conclusions will be of value when evaluating the application of certain species to native systems (Lopes et al., 2021) or predicting the response of communities when certain species are lost. If the definition of a keystone species includes those that are highly connected then keystone species may not always be those that are positive contributing members.

Previous work has shown in soil systems that diverse communities with redundancy are better able to respond to changes in the environment that may affect certain keystone members (Wagg et al., 2021). However, the results shown here suggest that even if diversity and redundancy for a particular pathway are present (i.e., chitin breakdown) communities may still not be able to survive the loss of a primary degrader keystone member if the new member filling this metabolic niche does not participate in the same sharing interactions. Our MSC-2 community had a great deal of redundancy related to chitin breakdown (5/8 species are able to metabolize chitin in monoculture (McClure et al., 2022)). Despite this, the communities’ response to keystone species loss (*Streptomyces*) was driven not by the fact that there was redundancy in the system but primarily by the nature of next emerging primary degrading species (*Rhodococcus*) and the fact that it may limit the exchange of resources with other community members.

Aside from interaction networks and the identity of primary chitin degraders, these results also show how CUE shifts in a set of communities across time as they cycle a complex C source such as chitin. Species lacking *Rhodococcus* show more rapid increases and decreases in respiration compared to the complete MSC-2 community. As described above this may be due to *Rhodococcus* not facilitating growth of MSC-2 as well as *Streptomyces* but it also allows us to see how CUE shifts as communities are cultured. At 178 h CUE levels suddenly increase in MSC-2-R communities with a drop in respiration and a large increase in biomass. The complete MSC-2 community also shows a large increase in biomass at this timepoint and its respiration levels appear to be peaking suggesting that, if the experiment has been continued, it would have matched MSC-2-R’s response with a drop in respiration. One possibility for this increase in CUE for both communities may be that the overall C pool is shifting as chitin is metabolized. In early timepoints much of the community may have been in a C starved and stressed state showing lower CUE (higher respiration and lower biomass accumulation; Schimel et al., 2007), since it appears that only a few species act as chitin degraders in MSC-2. However, as chitin is degraded (by *Streptomyces*, a sharing species) in the MSC-2 and MSC-2-R communities the C pool changes, and a number of new nutrients become available (N-acetyl-glucosamine and other chitin breakdown products). This removes the stress conditions for species and the CUE of the community as a whole increases as C is moved into a biomass accumulation pathway rather than a respiration/catabolic pathway. A more available C pool is supported by our previous work (McClure et al., 2022). These results suggest that changes in CUE can happen as a function of available carbon sources becoming more diversified and easier to metabolize by a greater number of community members. Shifts in C pools however, are a function of the species that are present. Metabolite profiles show that MSC-2-S communities show the least shift in their C pools compared to media only controls (Figure 4; equivalent to the carbon pools at the start of the experiment). This lack of shifting of C pools without *Streptomyces* is likely one reason why this community shows slower growth.

## Conclusion

We show here how a chitin degrading synthetic community of soil microbes, MSC-2, responds to loss of certain keystone members. Lack of a primary degrader (*Streptomyces*) causes a collapse in community growth and respiration even when other members have the genomic potential to fill the same metabolic niche. Even if diversity and redundancy are in place, if subsequent chitin degraders that step up in the absence of a primary degrader do not share metabolites, then the community cannot thrive. As soil microbiomes continue to shift in response to changing environments keystone species may bloom or die off and other species will likely emerge to replace them. The accepted paradigm is that diversity will allow for greater resilience due to the larger number of species available to fill required metabolic niches. While this is still true the results presented here show that simple redundancy of pathways is not enough. If the emerging species filling the metabolic niche does not possess the same sharing phenotype and interaction network, then the community will not grow as well. These results also show how synthetic communities can be used to better understand processes in native systems and will be of value when considering genomic redundancy in soil communities’ and how this gives the soil microbiome the ability to respond to changes that may take place over the next decades due to a changing climate.

## Data availability statement

All 16s data has been deposited to the PNNL DataHub, a public facing repository here: https://data.pnnl.gov/group/nodes/dataset/33294. Users must be login with an ORCID. All metabolomics data has been deposited in MASSIVE here https://massive.ucsd.edu/ProteoSAFe/dataset.jsp?accession=MSV000090720.

## Author contributions

RM, KH, and MG designed the experiments. MG and YF carried out the experiments and analyzed growth and respiration data. RM analyzed amplicon data. SC analyzed and interpreted metabolomics data. RM wrote the manuscript and KH led the overall project. All authors contributed to the article and approved the submitted version.

## Funding

This program is supported by the U. S. Department of Energy, Office of Science, through the Genomic Science Program, Office of Biological and Environmental Research, under FWP 70880. PNNL is a multi-program national laboratory operated by Battelle for the DOE under Contract DE-AC05-76RLO 1830. A portion of this work was performed in the William R. Wiley Environmental Molecular Sciences Laboratory (EMSL), a national scientific user facility sponsored by Office of Biological and Environmental Research and located at PNNL.

## Conflict of interest

The authors declare that the research was conducted in the absence of any commercial or financial relationships that could be construed as a potential conflict of interest.

## Publisher’s note

All claims expressed in this article are solely those of the authors and do not necessarily represent those of their affiliated organizations, or those of the publisher, the editors and the reviewers. Any product that may be evaluated in this article, or claim that may be made by its manufacturer, is not guaranteed or endorsed by the publisher.

## Supplementary material

SUPPLEMENTARY FIGURE S1 PCA of amplicon data from MSC-2 communities and subsets. Colors of samples are shown in the upper right. To remove differentially effects of the leave out aspect of the experiment this PCA includes amplicon data from all members of MSC-2 except for *Rhodococcus* and *Streptomyces*.

SUPPLEMENTARY FIGURE S2 PCA of metabolic from MSC-2 communities, subsets and media controls. Colors of samples are shown in the upper right.
