# Supplementary figures and images for: Removal of primary nutrient degraders reduces growth of soil microbial communities with genomic redundancy
Source: Front Microbiol. 2023 Jan 24;13:1046661. doi: 10.3389/fmicb.2022.1046661 (PMC9902710; doi:10.3389/fmicb.2022.1046661)

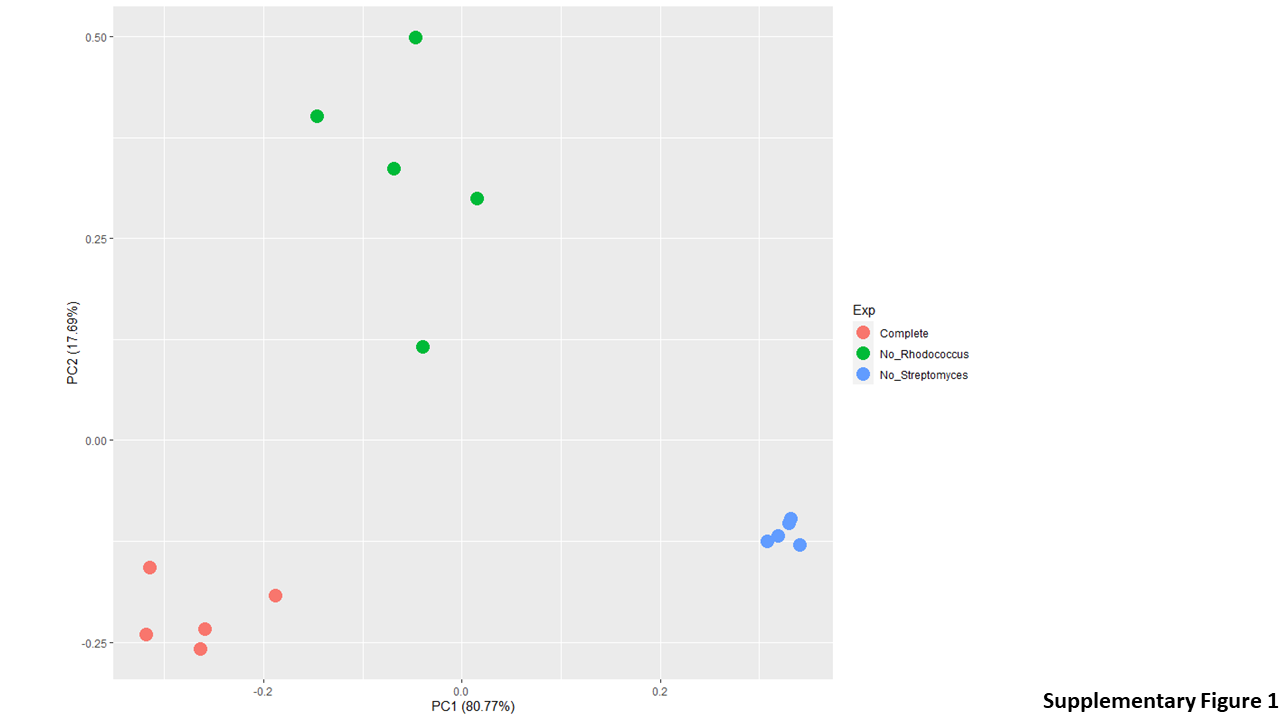

Supplement: SUPPLEMENTARY FIGURE S1 — PCA of amplicon data from MSC-2 communities and subsets. Colors of samples are shown in the upper right. To remove differentially effects of the leave out aspect of the experiment this PCA includes amplicon data from all members of MSC-2 except for Rhodococcus and Streptomyces. [file Image_1.TIF]

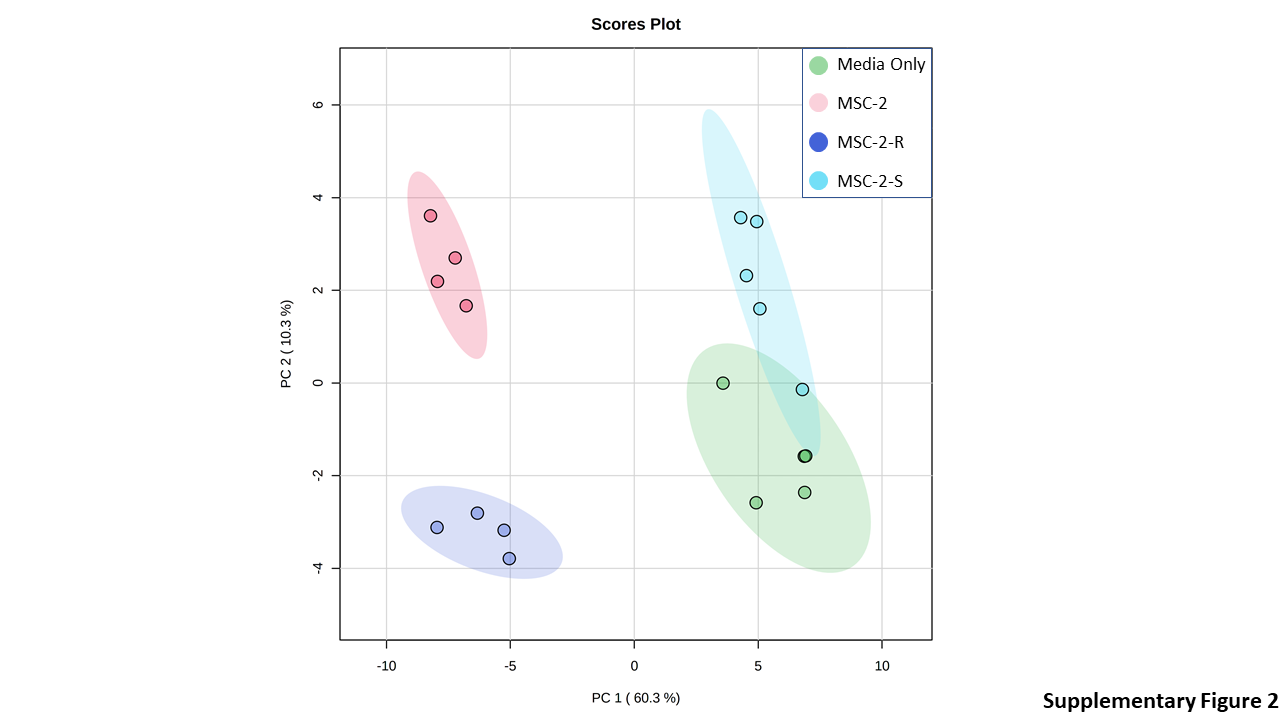

Supplement: SUPPLEMENTARY FIGURE S2 — PCA of metabolic from MSC-2 communities, subsets and media controls. Colors of samples are shown in the upper right. [file Image_2.TIF]
